# Scale-Up of Tailor-Made Onsite Enzyme Blend From Cassava Peels for Industrial Bioethanol Production

**DOI:** 10.1155/tswj/2296078

**Published:** 2025-01-21

**Authors:** Martison Budu, Patrick Boakye, Joseph A. Bentil

**Affiliations:** ^1^Department of Biochemistry and Biotechnology, College of Science, Kwame Nkrumah University of Science and Technology, Kumasi, Ghana; ^2^Department of Chemical Engineering, College of Engineering, Kwame Nkrumah University of Science and Technology, Kumasi, Ghana

**Keywords:** bioethanol, cassava peels, cassava-degrading fungi, onsite enzyme, optimization, substrate

## Abstract

Bioethanol production is one of the key alternatives for fossil fuel use due to climate change. The study seeks to upscale tailor-made onsite enzyme blends for the bioconversion of cassava peels to bioethanol in simultaneous saccharification and fermentation (SSF) process using cassava peels-degrading fungi. The starch and cellulose contents of peels were determined. The results showed significant levels of cellulose (39.78%) and starch (31.21%), indicating that cassava peels are valuable raw materials for bioethanol production. To investigate cassava-degrading microbes, *Aspergillus niger* demonstrated the highest enzyme activity with a diameter of zone of clearance of 16 mm. Tailor-made enzyme blends were produced with the *A*. *niger* using various substrate concentrations (1%, 3%, 5%, 8%, and 10%) of milled cassava peels at periods of 2, 4, 6, 8, and 10 days with a spore concentration of 2.1 × 10^5^ cells/mL. The amylolytic and cellulolytic activities were determined as 4.759 U/mL and 4.265 U/mL, respectively, at 5% and 6-day optimal conditions. The enzyme blend was upscaled using three fermentation vessels, thus 0.250 L flask, 1.0 L flask, and 10 L fermenter at optimized conditions in the SSF process for bioethanol production. These optimal conditions were firstly applied to a 0.250 L flask in the SSF process, a fixed enzyme dose of 20 mL and 1.5 g of *Saccharomyces cerevisiae* with varying substrate concentrations of 5%, 10%, and 20% and ethanol analyzed daily for 10 days. The theoretical ethanol yields recorded were 15.64%, 16.02%, and 14.91% for 0.250, 1, and 10 volumes obtained at different fermentation days. These optimized conditions demonstrate the potential for industrial bioethanol production in Ghana.

## 1. Introduction

The focus of the United Nations' sustainable development goal number 13 is to address climate change, as its severe impact is affecting every country worldwide. Greenhouse gas emissions have increased by over 50% since 1990, and 2019 marked the second warmest year and the end of the warmest decade ever recorded [1]. Carbon dioxide (CO_2_) and other atmospheric greenhouse gas levels reached new records in 2019 [1, 2]. Using fossil fuels without measures to reduce greenhouse gas emissions contributes to global climate change. It is evident that enhancing energy efficiency and promoting renewable energy sources is crucial for mitigating climate change and reducing risks to ecosystems [1].

The climate change effect has led to increasing global ethanol production, but it experienced a decline in 2020 due to the COVID-19 pandemic. The United States is the largest producer of ethanol, followed by Brazil, the European Union, China, and Canada [3]. The rest of the world also generated a significant amount of ethanol [3]. In Africa, where many countries are net oil importers, there is a growing need to develop alternative fuels for energy self-sufficiency and socioeconomic benefits [4, 5]. Biofuels are seen as a potential solution, and governments worldwide have implemented policies to promote their use and reduce CO_2_ [6]. In Ghana, there have been attempts to establish biofuel policies, such as using biodiesel blends for government vehicles and increasing the production of Jatropha and cassava/sugarcane for bioethanol [7–9].

Bioethanol is a leading biofuel that has experienced significant market growth [10]. It is produced by fermenting plants and biomass materials' starch and sugar components [11, 12]. Compared with other generations of bioethanol production, this study focuses on second-generation bioethanol, which utilizes lignocellulosic biomass. Lignocellulose found abundantly in both woody and nonwoody plants is the most common renewable energy source globally and a byproduct of various plant processing materials [6, 11]. Its primary components are cellulose, hemicellulose, and lignin. The use of second-generation bioethanol offers numerous advantages over other generations due to the widespread availability and sustainability of lignocellulosic biomass [13, 14]. Cassava (*Manihot esculenta*) is a starchy root crop commonly used as a food source in developing nations [15]. It is extensively cultivated in tropical and subtropical regions of Asia, South America, and Africa such as Ghana [15].

Cassava peels are an ideal feedstock for bioethanol production due to their low lignin content, which facilitates the separation of fermentable sugars for bioethanol processing. Moreover, their abundant availability and affordability make them a viable option [16–18].

Fungi are widely recognized as effective microorganisms for breaking down lignocellulose into its main components [19]. Hydrolytic enzymes, such as cellulases and amylases, are crucial for efficient hydrolysis of cellulose and starch, respectively. Some fungi, particularly species of Aspergillus, Rhizopus, and Penicillium, are known to produce these enzymes abundantly [20]. *Aspergillus niger* is highly regarded for its enzyme production capabilities and is considered safe for use in various biotechnological applications by the US FDA [21, 22]. Enzymes play a significant role in the cost of bioethanol production and producing them onsite can help reduce expenses [23]. Among the enzyme-catalyzed approaches, simultaneous saccharification and fermentation (SSF) is preferred due to its ability to achieve higher yields and concentrations of ethanol [13, 24]. This method combines enzymatic cellulose hydrolysis with the simultaneous fermentation of glucose to ethanol, resulting in improved efficiency and cost-effectiveness [25]. The SSF technique also avoids the need for expensive equipment and minimizes the risk of contamination [26, 27]. The use of yeast in the enzyme complex helps enhance yields and saccharification rates by reducing the accumulation of inhibitory sugars in the reactor [28]. The objective of this research is to scale up the production of a tailor-made onsite enzyme blend from cassava peels to produce bioethanol through SSF processes.

## 2. Materials and Methods

### 2.1. Location of Study

The present study was conducted at the laboratories of the Department of Biochemistry and Biotechnology and the Department of Chemical Engineering all in Kwame Nkrumah University of Science and Technology (KNUST), Kumasi, in the Ashanti region of Ghana.

### 2.2. Sources of Experimental Materials

The feedstock of cassava peels that served as substrate was procured from the Gari processing facility at Asuboi in the Ayesuano district close to Suhum on the Accra-Kumasi Highway in the Eastern Region of Ghana. From the same cassava processing factory's disposal site, two more samples of fresh cassava peels (FCPs) and decayed cassava peels (DCPs) for fungal isolation were collected.

### 2.3. Sample Preparation

The cassava peels used as feedstock were processed in a series of steps. First, they were washed with water to remove any unwanted particles and dust. Then, the peels were dried in the sun for 7 days to eliminate moisture. Once dried, the cassava peels were milled, placed in a sealed poly bag, and stored for future studies. In addition, fresh and partially DCPs were treated the same way.

### 2.4. Chemical Analysis of Cassava Peels

This was done in the Nutrition Laboratory of the Animal Science Department, KNUST. The insoluble fiber in cassava peel, which are cellulose, hemicellulose, and lignin contents, was determined as acid detergent fiber (ADF), neutral detergent fiber (NDF), and acid detergent lignin (ADL) according to the method of analysis used by Dubey et al. [29]. The starch analysis from the cassava peels was also conducted using the wet method described by [30].

### 2.5. Isolation of Fungi

The isolation of the fungi from the FCPs and the DCPs was undertaken at the Soil Science Laboratory of KNUST. The investigation employed the simple dilution plate method. Each sample, weighing 1 g, was diluted three-fold in 9 mL of sterile distilled water. The final dilution (10^−3^) was then flooded onto three separate sterile potato dextrose agar (PDA) medium plates that were supplemented with chloramphenicol antibiotic to inhibit bacterial growth. The plates were incubated at 28°C for 7 days. The resulting fungal growth was counted using a colony counter and identified using the Illustrated Genera of Imperfect Fungi Manual by [31]. The frequencies of occurrence for each identified fungus were determined. Subsequently, the cultures were subcultured until pure isolates were obtained.

### 2.6. Macromorphological and Micromorphological Characteristic Identification

The fungal isolates were identified based on their macromorphological and micromorphological characteristics. For macromorphological identification, colony traits such as color, texture, and spore structure were observed, following the guidelines provided in the handbook for the identification of fungi [31]. Micromorphological characteristics were examined using the Conventional Lactophenol Cotton Blue Technique (LPCB). Slides for microscopic analysis were prepared using five-day-old pure cultures. A small number of mycelia was placed on a slide, and a drop of lactophenol blue was added. A cover slip was then applied, and the sample was examined under a light microscope at a magnification of 400×. Identification was conducted by comparing the observed features with relevant micrographs. The fungi obtained were identified as *Aspergillus niger*, *Aspergillus flavus*, and *Rhizopus stolonifera*.

### 2.7. Qualitative Analysis of the Three Fungal Isolates for Enzyme Activity

The three fungal isolates were cultivated on a PDA medium. Subsequently, the isolates were tested for enzyme activity by incorporating 3 g of cassava peels into a PDA medium. Three separate media were prepared, each inoculated with one of the three fungi and then incubated at 30°C. An iodine solution containing iodine (0.2 mL), potassium iodide (0.4 mL), and distilled water (100 mL) was flooded onto each medium. The presence of a clear zone around the fungal growth indicated the production of amylase. To identify cellulase enzymes, the plates were flooded with a 1% Congo red solution and left at 28°C for 20 min. Subsequently, the plates were thoroughly rinsed with a 1M sodium chloride solution. The formation of a clear zone surrounding the cellulase-producing colonies against a dark red background indicated cellulase activity.

### 2.8. Preparation of *A*. *niger* Spore Suspension

After incubation for 5 days, the fungal spores were harvested by saturating the culture with 10 mL of sterilized distilled water and then scraping the spores out. The spore suspensions were then decanted and combined to the appropriate volume in a 100 mL Erlenmeyer flask.

### 2.9. Determination of Spore Concentration

One milliliter of spore suspension was transferred from the stock into a measuring cylinder with a capacity of 100 mL. After shaking the diluted sample, a portion was pipetted into the grooves of the hemacytometer and placed under a light microscope to count the spores. The average of the spore counts from the hemacytometer five zones was calculated. The average value was then multiplied by a dilution factor. The spore concentration was determined as 2.1 × 10^5^ spores/mL.

### 2.10. Preparation of Growth Medium

Basal salt medium (BSM) was prepared according to the combinations used by [32]with slight modifications. The BSM containing 1.4 g of (NH_4_)_2_SO_4_, 0.3 g of MgSO_4_.7H_2_O, 2.0 g of KH_2_PO_4_, 0.5 g of FeSO_4_, 0.3 g of CaCl_2,_ 0.3 g of urea, 1 mL of Tween 20, 0.16 g of MnSO_4_, 0.14 g of ZnSO_4_, 0.2 g of CoCl_2_, and 10 g of starch in 1 L of deionized water was prepared. No additional carbon or nitrogen source was added. The pH of the medium was adjusted to 5.5.

### 2.11. Enzyme Production (*α*-Amylase and Cellulase) From *A*. *niger*

Temperature and pH were the fixed conditions maintained during the enzyme production. The optimum temperature and pH were used in this research. For the enzyme production by *A*. *niger*, an optimum temperature of 30°C and a pH of 5.5, respectively, were used. These fixed conditions are within the range for optimum production of amylase and cellulase enzymes as reported in the literature for *A*. *niger* [21].

Substrate concentration and incubation time, two essential variables, were varied in this study. Varying amounts of 1%, 3%, 5%, 8%, and 10% (w/v) of the dry-weight biomass of the cassava peels were used as the substrate concentrations. On the other hand, the incubation time was varied as 0, 2, 4, 6, 8, and 10 days. It was essential to do this to obtain the ideal substrate concentration and incubation time for bioethanol production.

The five distinct substrate concentrations, 1%, 3%, 5%, 8%, and 10% (w/v) of the cassava peels, were added in triplicate to five different 50 mL flasks that contained 25 mL of culture media. The flasks and their contents were autoclaved for 15 min at 121°C and were then cooled to room temperature. After that, the flasks were inoculated with 5 mL of spore suspension of *A*. *niger* containing 2.1 × 10^5^ spores/mL, and they were incubated in an orbital shaker at 150 rpm and 30°C for the indicated varied number of days: 0, 2, 4, 6, 8, and 10 days. Amylase and cellulase activity tests were performed after samples were collected at the specified time intervals.

### 2.12. Recovery of the Crude Enzyme Extract (Tailor-Made Enzymes)

At various periodic intervals indicated, samples were taken from the flasks and filtered. The filtrate which contains the crude enzymes was recovered through centrifugation at 7000 rpm for 30 min at 30°C. The supernatant was then decanted into a test tube for further analysis.

### 2.13. Measurement of Enzyme Activity

The amylase and cellulase assays were performed using the dinitrosalicylic acid (DNSA) technique [32, 33]. For the amylase activity measurement, 0.5 mL of soluble starch solution and 1 mL of potassium phosphate buffer (pH 6.8) were mixed in five separate test tubes. Similarly, for cellulase activity measurement, 0.5 mL of CMC solution and 1 mL of sodium acetate buffer (pH 5.5) were mixed in five independent test tubes. To initiate the reaction, 0.5 mL of the raw enzyme extract was added to each mixture. The tubes were then incubated at 50°C for 30 min. All five samples, which were collected at regular intervals for examination for 10 days, followed this procedure. A control test tube was also prepared for both assays. After the incubation period, 1 mL of DNS reagent was added, and the reaction was stopped by placing all the tubes in a boiling water bath at 95°C for 5 min. The amount of reducing sugar released (glucose) was determined by measuring the absorbance at 540 nm using a spectrophotometer with the DNS technique. The concentration of reducing sugars was calculated by comparing the results with a standard glucose curve. In this context, one unit of amylase and cellulase activity is defined as the amount of enzyme that can release 1 µmole of reducing sugars per minute under the specified assay conditions [34].

### 2.14. Optimization of Tailor-Made Enzymes

The optimum conditions obtained in the outcome of the initial enzyme extraction process as outlined earlier were used to scale up the enzyme extraction in a 500 mL flask. The initial experiment results proved that 10% substrate concentration and a 4-day period were the optimum variable conditions for enzyme extraction in this experiment as a temperature of 30°C and pH of 5.5 remains constant with the same already prepared BSM. The DNSA method was used for the amylase and cellulase enzyme assays. The absorbance at 540 nm was measured against a standard curve as was done in the initial process. The crude enzyme extract (CEE) was stored for the hydrolysis process.

### 2.15. Bioethanol Production

#### 2.15.1. SSF

The crude tailor-made enzyme blend (amylase and cellulase) produced was used onsite to test for ethanol production efficiency by flask fermentation in order to get the optimum conditions for the scale-up of the final bioethanol production in a 10 L bioreactor. This initial experimental process was done in shake flasks (250 mL). An enzyme dose of 20 mL, a temperature of 50°C, and a pH of 5.0 were maintained as fixed conditions throughout the experimental period. Substrate concentrations of 5%, 10%, and 20% (w/v) were varied in the saccharification process.

Three different conical flasks were utilized for the given substrate concentrations of 5%, 10%, and 20% w/v. In each of the three conical flasks, the already prepared cassava peel flour was dissolved in an initial volume of distilled water. To reach the target, more water was poured into the flask. The flasks were sealed with cotton wool, vigorously shaken, and then autoclaved at 121°C for 15 min. After that, 20 mL of the enzyme mixture which contains the crude enzyme blend was added to each flask as an inoculant, and brewer's yeast (obtained from Anchor Yeast, South Africa) with a concentration of 1.5 g was added to the fermentation broth. Each conical flask's mixture was sealed with aluminum foil and kept under anaerobic conditions for 7 days. The optimized conditions were then used in the scale-up process using 1 L and 10 L fermentation vessels. After each period, the fermented was filtered and the ethanol concentration was determined using gas chromatography (GC).

#### 2.15.2. GCAnalysis

The ethanol contents were detected qualitatively and quantitatively with the Shimadzu GC-2010 plus (Shimadzu Scientific Instruments, Columbia, USA) GC and an AutoSampler (AOC 20 s). The GC instrument had a capillary column VF-5 Ms (5% phenyl and 95% dimethylpolysiloxane) of 30 m length, 0.25 mm inner diameter, and 0.25 μM film thickness. An aliquot of 1 μL sample was injected into the GC with an Auto-Injector AOC 20i, keeping the injector temperature at 240°C. The injection mode was set to the split mode with a split ratio 100:1. Nitrogen gas (99.9%) was used as a carrier gas at a constant flow rate of 1 mL/min. The column temperature was initially set at 40°C (hold time, 2 min), increasing 15°C/min to 100°C (hold time, 1 min) and a total program time of 7 min. The flame ionization detector (FID) was operating at 250°C during the analysis. The data were then retrieved for further processing.

### 2.16. Statistical Analysis

All analyses were done in triplicates and results were presented as mean standard values. One-way analysis of variance (ANOVA) was used for the comparison of means using Microsoft Excel 365 ProPlus. The significance of *p* < 0.05 was accepted.

## 3. Results and Discussion

The data obtained were extracted from an earlier publication in preprint [35].

### 3.1. Lignocellulosic and Starch Characterization

Table 1 indicates the parameters of the percentage insoluble portion of the cassava peels and that of starch. At 0.5 g of the total cassava peels analyzed for the insoluble portion, the composition of cellulose was the highest (39.78%) and the lignin content was found to be the lowest (3.84%). There was also a 31.21% starch determined according to the protocol as described by [34].

Cassava peels are utilized as a raw material in second-generation bioethanol production, as they can be hydrolyzed to produce crucial sugars required for bioethanol production. Understanding the initial levels of these constituents in the materials used is important. In this experiment, the dried cassava peel sample was analyzed using the NDF, ADF, and ADL methods to determine its lignocellulosic composition. Table 1 presents the percentage composition of cassava peels, which includes starch (31.21%), cellulose (39.78%), hemicellulose (21.11%), and lignin (3.84%). Similar results were reported by [5] for cellulose (40.5%) and hemicellulose (21.4%), but there was a significant difference in the lignin content (11.7%). Conversely, the authors in [36] reported lower cellulose content (11.30%) and similar hemicellulose and lignin contents (22.39% and 3.09%, respectively). Reference [10] reported a wider range of starch content in their experiments (47.16% and 15.82%, respectively). The results of the current experiment, as shown in Table 1, highlight two important characteristics of cassava peels that make them suitable for bioethanol production. First, they have a relatively high composition of cellulose and starch, which, when properly hydrolyzed, can yield significant amounts of glucose and maltose. Second, the biomass has a low lignin content, which reduces the recalcitrance of the lignocellulosic material during enzymatic pretreatment.

Microorganisms, including bacteria and fungi present in the environment, play a crucial role in the degradation of cassava peels. The presence of fungal organisms in the soil, particularly in gari processing sites, aligns with a report by [37], indicating that soil serves as a reservoir for plant-degrading organisms. This research focused on the isolation of fungi for microbial analysis.  Table 2 presents the results of fungal isolation from two different samples obtained from the same source. In FCPs, only one type of fungal isolate, identified as *A*. *niger*, was found. In DCPs, three different fungi were identified, namely, *A*. *niger*, *A*. *flavus*, and *R*. *stolonifer*. Previous studies conducted by [38] reported the presence of *A*. *niger* and *Rhizopus* spp. in the fungi isolated from cassava peels whereas [37] recorded *A*. *niger* and *A*. *flavus* as part of the fungi they isolated from cassava peels.  Table 3 demonstrates the clear degradative abilities of the isolated fungi, with all three isolates showing evidence of enzyme production. Among the three isolates, *A*. *niger* exhibited the largest zone of clearance around the colony, indicating its high enzyme production capability.

### 3.2. Qualitative Analysis of the Isolates for Enzyme Production

All three isolates were screened for amylase and cellulase production using the PDA plate method resulting in a clear zone of enzyme hydrolysis in the Petri dishes after treatment with iodine and Congo red solutions. The zone of clearance analysis of all the isolates as indicated in Table 3 revealed that on average, *A*. *niger* got the highest zone of clearance while *A*. *flavus* obtained the least. *A*. *niger* was then chosen and preserved for further analysis.

A crude enzyme blend was generated using cassava peels as a substrate in a 250 mL flask. The presence of an enzyme cocktail in the crude extract was confirmed by assessing the activity of amylase and cellulase enzymes. These enzymes belong to a group that breaks down complex sugars into simpler forms. Amylase and cellulase play a vital role in the saccharification process of various natural substrates for biofuel production, as highlighted by [32, 34]. The cost of enzymes constitutes a significant portion (10%–20%) of the overall expenses involved in producing ethanol from biomass. This financial aspect has prompted numerous studies to explore ways to reduce enzyme costs in bioethanol production. One suggested approach is the onsite production of economical lignocellulosic raw materials, which can help alleviate the financial burden associated with enzyme procurement [8]. In line with this, the present study aims to address this cost-efficiency concern by utilizing cassava peels for the onsite production of a crude enzyme blend. This approach offers a viable and economical means of obtaining enzymes for bioethanol production.

**Figure 1:**
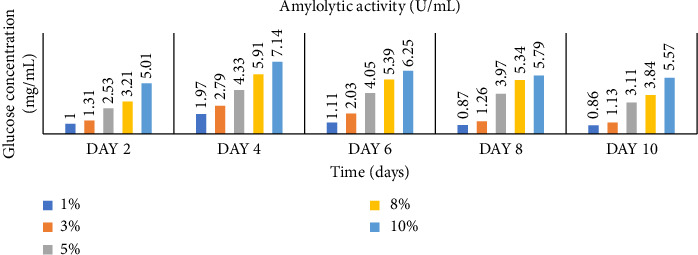
Glucose concentration (mg/mL) produced at varying concentrations of starch with time (days).

**Figure 2:**
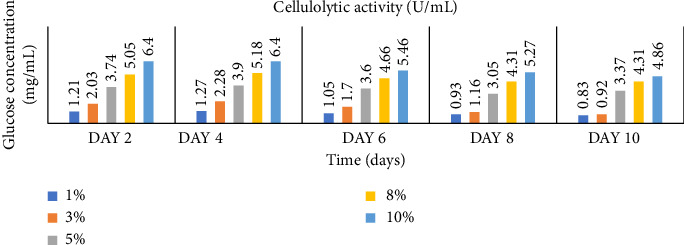
Glucose concentration (mg/mL) produced at varying concentrations of cellulose with time (days).

**Figure 3:**
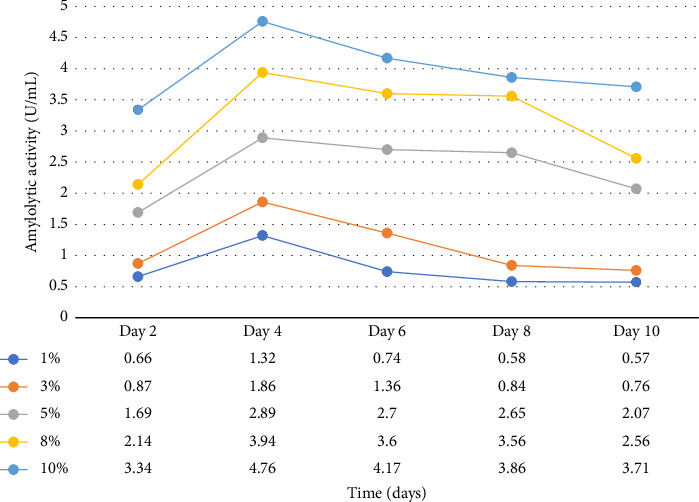
Amylolytic activities at varying substrate concentrations and time (days).

**Figure 4:**
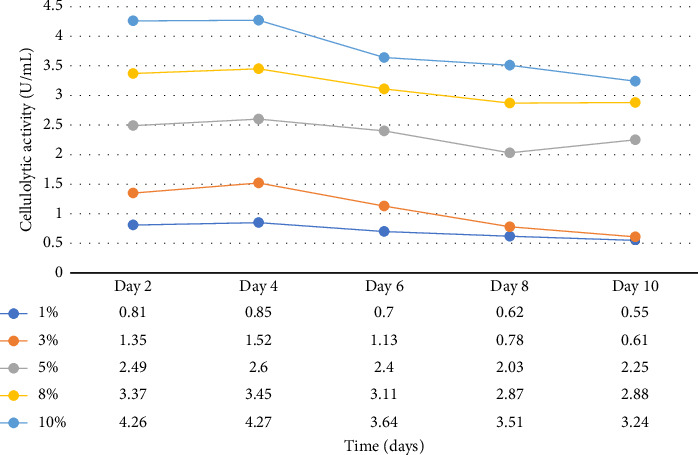
Cellulolytic activities at varying substrate concentrations and time (days).

Enzymatic activity can be measured using two approaches: assessing the remaining amount of substrate or quantifying the produced product resulting from the enzyme-catalyzed reaction. In the case of amylase and cellulase, the enzymatic reactions yield glucose. To measure the product concentration, a colorimetric assay was employed. This involved determining the absorbance and utilizing these values to calculate the corresponding product concentrations, as shown in Figures 1 and 2. Finally, the enzyme activities (amylases and cellulases) were determined and reported in Figures 3 and 4. The results of the amylolytic and cellulolytic activities measurements using starch and carboxymethyl cellulose (CMC) yielded values of 4.76 U/mL and 4.27 U/mL, respectively. These results demonstrate that both amylase and cellulase exhibited their highest enzyme activity on day 4 with a substrate concentration of 10%. This finding is consistent with the research conducted by [24], who also reported the highest enzyme activity on day 4.

**Figure 5:**
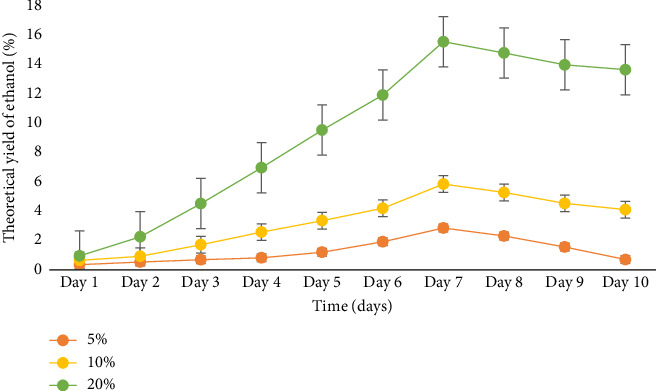
The theoretical yield of ethanol produced from different substrate concentrations at varying periods in a 0.25 L flask.

The production of ethanol is closely linked to the growth of beneficial microbial cells. According to [22, 28], the number of yeast cells and the amount of substrate have a direct impact on ethanol production. In this study, the enzymatic production of bioethanol from cassava peels using *A*. *niger* was examined. The fermentation process involved the utilization of Brewer's yeast (*S*. *cerevisiae*). The results presented in Figure 5 demonstrate that a substrate concentration of 20% and a fermentation period of 7 days gave the highest ethanol yield of 15.64% from cassava peels in 250 mL flask fermentation. Notably, the percentage of ethanol production showed a significant increase for all samples during the initial 7 days of fermentation. Day 1 exhibited the lowest ethanol levels, while Day 7 showed the highest levels. After Day 7, a gradual decline in ethanol concentration was observed. Reference [12] explained that the initial surge in ethanol levels can be attributed to the presence of sufficient fermentable sugars, allowing yeast to efficiently convert these sugars into ethanol through digestion.

The subsequent decrease in ethanol levels indicates the depletion of available fermentable sugars necessary for yeast growth. The use of *A*. *niger* for cassava peel hydrolysis and *S*. *cerevisiae* for bioethanol production has been reported as effective according to [16, 31]. On the contrary, lower yields of ethanol (1.91%; 1.89%) were reported by [26, 39] in 100 mL flasks, which were significantly lower than the 15.64% yield obtained in this study. In addition, the authors in [3] recorded higher ethanol concentrations of 42.6% and 38.7% for other cassava cultivars and 20.5% for cassava peels on day 6, which exceeded the results of the current study.

**Figure 6:**
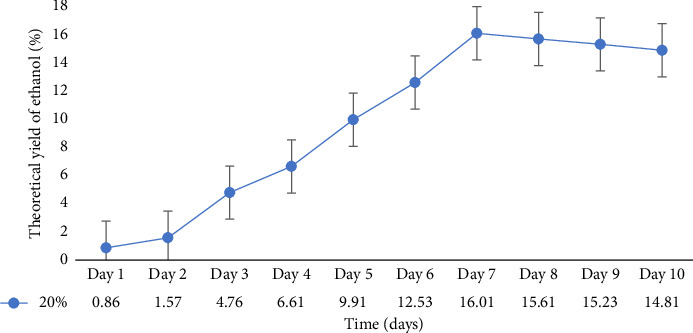
The theoretical yield of ethanol from 20% substrate concentration optimum at varying periods in a 1 L flask.

**Figure 7:**
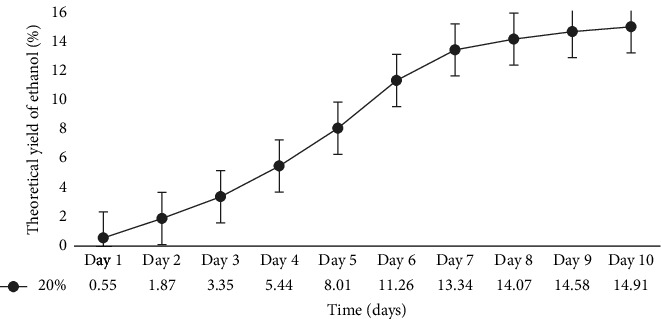
The theoretical yield of ethanol from 20% substrate concentration optimum at varying periods in a 10 L fermenter.

In a flask experiment conducted by [16] for 5 days using mixed cultures, 50 g of cassava peel yielded 11.97 g/cm^3^ (26.0%) ethanol. However, when only *Z*. *mobilis* or *S*. *cerevisiae* were used for the separate fermentation process, the mass of bioethanol produced from cassava peels was 10.6 g/cm^3^ (23%). Furthermore, with 35 g of substrate, cassava peel had a yield of 9.64 g/cm^3^ (20%), and with 20 g of substrate, it had a yield of 7.8 g/cm^3^ (14%). In a 500 mL flask experiment conducted by [11] for 21 days, an ethanol concentration of 14.46 g/cm^3^ (38%) was obtained from cassava peels, which exceeded the results of the current study. Similarly, the authors in [40] recorded a 15% ethanol concentration when 50 g of cassava peels was used in a 500 mL flask for 7 days. The primary objective of the present study is to scale up the flask experiment using the optimized conditions obtained. Previous research on bioethanol production from cassava peels primarily focused on flask experiments with volumes ranging from 50 mL to 500 mL and durations ranging from hours to 10 days. In the context of Ghana, there has been limited investigation on the use of cassava peels for bioethanol production. To scale up this study, a 10 L bioreactor was employed. To ensure the consistency of the optimized conditions, a 1 L fermentation was performed (Figure 6). According to the graph, the optimal ethanol production of 16.02% was achieved on Day 7, which was consistent with the results from the 0.250 L flask fermentation. However, the result from the10 L bioreactor (Figure 7) yielded an ethanol concentration of 14.91% on Day 10.

This ethanol concentration was slightly lower compared with the results from both the 0.250 and 1 L fermentation processes. This difference may be attributed to various factors such as environmental conditions, agitation levels, homogeneity, and the means of CO_2_ liberation during fermentation. In a previous study by [41], a 5 L bioreactor scale up yielded an ethanol concentration of 1.48% after 72 h, which was lower in both time and value than the results obtained from flask fermentation in the current study. On the contrary, the authors in [39] achieved a higher ethanol concentration of 35.95% in 2.21 L fermentation volume in just 1 h and 50 min from 4.85 kg of cassava peels using a constructed industrial ethanol production plant.

## 4. Conclusion

Based on the findings of the present study, several conclusions can be drawn. First, cassava peel is a renewable resource and a suitable substrate for bioethanol production. Second, the onsite production of a crude enzyme cocktail from cassava peel using *A*. *niger* proves to be an efficient method for obtaining enzymes required for the hydrolysis of the constituents of cassava peels, thus cellulose and starch. This approach offers economic benefits in the production process. Lastly, the scale-up fermentation using SSF indicates that a substrate concentration of 20% cassava peel, along with 1.5 g of *S. cerevisiae* and 20mL of CEE, is suitable for bioethanol production. These optimized conditions demonstrate the potential for industrial bioethanol production in Ghana.

## Figures and Tables

**Table 1:** Lignocellulosic and starch composition (%) of cassava peels (methods described by [34]).

Cassava peel composition	Percentage of dry cassava peel components (%)
Cellulose	39.78 ± 0.97
Hemicellulose	21.11 ± 0.81
Lignin	3.84 ± 0.07
Starch	31.21 ± 0.93

**Table 2:** Fungi isolates from different sources of cassava peels.

Sample sources	Spore plate counts^∗^		
	*Aspergillus niger*	*Aspergillus flavus*	*Rhizopus stolonifera*
FCP	1	0	0
	0	0	0
	1	0	0

DCP	0	6	1
	1	1	0
	0	2	0

Abbreviations: DCPs, decaying cassava peels; FCPs, fresh cassava peels.

∗Figures represent colony counts for isolated fungi.

**Table 3:** Hydrolytic activities of fungal isolates on cassava peels.

Sample	Diameter of hydrolytic zone (mm)
*Aspergillus niger*	16 ± 0.75
*Aspergillus flavus*	9 ± 0.61
*Rhizopus stolonifera*	13 ± 0.82

## Data Availability

The data used to support the findings of this study are available from the corresponding author upon reasonable request.
